# Plasma metabolites were associated with spatial working memory in major depressive disorder

**DOI:** 10.1097/MD.0000000000024581

**Published:** 2021-02-26

**Authors:** Yue Du, Jinxue Wei, Xiao Yang, Yikai Dou, Liansheng Zhao, Xueyu Qi, Xueli Yu, Wanjun Guo, Qiang Wang, Wei Deng, Minli Li, Dongtao Lin, Tao Li, Xiaohong Ma

**Affiliations:** a Psychiatric Laboratory and Mental Health Center; b West China Brain Research Center, West China Hospital of Sichuan University, Chengdu; c College of Foreign Languages and Cultures, Sichuan University, PR China.

**Keywords:** major depressive disorder, metabolomics, spatial working memory

## Abstract

Major depressive disorder (MDD) is a common disease with both affective and cognitive disorders. Alterations in metabolic systems of MDD patients have been reported, but the underlying mechanisms still remains unclear. We sought to identify abnormal metabolites in MDD by metabolomics and to explore the association between differential metabolites and neurocognitive dysfunction.

Plasma samples from 53 MDD patients and 83 sex-, gender-, BMI-matched healthy controls (HCs) were collected. Liquid chromatography-tandem mass spectrometry (LC-MS/MS) system was then used to detect metabolites in those samples. Two different algorithms were applied to identify differential metabolites in 2 groups. Of the 136 participants, 35 MDD patients and 48 HCs had completed spatial working memory test. Spearman rank correlation coefficient was applied to explore the relationship between differential metabolites and working memory in these 2 groups.

The top 5 metabolites which were found in sparse partial least squares-discriminant analysis (sPLS-DA) model and random forest (RF) model were the same, and significant difference was found in 3 metabolites between MDD and HCs, namely, gamma-glutamyl leucine, leucine-enkephalin, and valeric acid. In addition, MDD patients had higher scores in spatial working memory (SWM) between errors and total errors than HCs. Valeric acid was positively correlated with working memory in MDD group.

Gamma-glutamyl leucine, leucine-enkephalin, and valeric acid were preliminarily proven to be decreased in MDD patients. In addition, MDD patients performed worse in working memory than HCs. Dysfunction in working memory of MDD individuals was associated with valeric acid.

## Abstract 2

Supplemental Digital Content is available in the text

## 1 Introduction

Major depressive disorder (MDD) is a severe psychiatric disorder with affective and cognitive disturbance.^[1]^ In recent years, MDD has affected more than 300 million people around the world, and it had been one of the leading factors for the global burden of disease.^[2]^ Studies demonstrated that the longer duration of untreated depression could lead to higher rate of suicide and relapse, and early recognition and treatment are the key to this problem.^[3,4]^ However, the unknown etiology and pathogenesis of MDD contribute to high diagnostic error rates in primary care.^[5]^ Hence, there is an urgent need to investigate the underlying pathophysiological mechanism of MDD.

Previous researches suggested that metabolic dysfunction in MDD patients may induce the onset of this disease.^[6]^ The dysfunction of hypothalamic-pituitary-adrenal (HPA) axis in MDD has been widely investigated.^[1]^ Accumulating evidence demonstrated that the overactivity of HPA axis could lead to both depressive and cognitive disorder,^[7]^ and dysfunction in this axis was also related to metabolic syndrome in MDD patients.^[8]^ Previous studies showed that we could even find alterations in metabolic systems of MDD patients when first diagnosed.^[6]^ A review highlighted that dysfunction of metabolic processes involving energy metabolism and amino acid metabolism etc. were identified by using metabolomics method with samples from cerebrospinal fluid, blood, and urine of MDD patients.^[9]^ Metabolomics, which is a novel detection technology of the global metabolic response of multiple living systems, has recently been widely used for making a thorough inquiry of the metabolic alteration in psychiatric disorders.^[10]^ A large simple size research involving 5,\283 MDD patients and 10,145 controls indicated that 21 metabolites were significantly related to the pathogenesis of depression, and that these metabolites may contribute to the complication of cardiometabolic disorders in MDD patients.^[11]^ A meta-analysis of peripheral blood metabolites indicated that over 249 metabolites had been significantly different between MDD patients and healthy controls (HCs), and at least 106 metabolites were repeatedly identified in different samples.^[9,12]^ Nevertheless, those identified metabolites had limitedly clinical applicability for a number of reasons such as methodological differences and small sample sizes.^[13]^

Following the advances in metabolomics research, more and more analytic methods have been created to solve the problems aforementioned, including principal components analysis, partial least squares-discriminant analysis (PLS-DA), and so on.^[14,15]^ Nowadays, more advanced modeling has been further studied. Sparse partial least squares-discriminant analysis (sPLS-DA) is an under-supervised method for classification. It is a natural extension of PLS-DA, but it can perform better in large data sets, especially those with a large number of biological features (usually thousands) and small sample sizes (usually less than 50).^[16,17]^ In addition, the machine-learning method random forest (RF) analysis that uses an ensemble of selection trees has been suggested as an ideal model for feature classification from small samples.^[18]^ This method has also shown superior characteristics for analyzing large metabolomics datasets.^[19]^ Both sPLS-DA and RF analysis can help to find the differential metabolites in MDD, and to explain the mechanisms of metabolic alterations in this disease.

Cognitive disturbance is also a core symptom of affective disorder.^[20]^ MDD patients have plenty of neuropsychological disorders,^[21]^ including sustained attention, memory and executive function.^[22]^ Increasing literatures have suggested that working memory involving executive function plays a critical role in the whole process of MDD, and the performance in spatial working memory task of experimenters is also sensitive to structural change in frontal lobe damage which is also related to affective dysfunction.^[23,24]^ Previous researches have indicated that MDD patients have significantly worse working memory than HCs,^[25]^ and this deficit can induce seriously poor function in academic, occupational, and interpersonal realms.^[26]^ In addition, dysfunction in working memory can also be a typical feature of depressive patients, even who have mild symptoms of depression only.^[27]^Furthermore, metabolic dysfunction can also affect working memory function.^[28]^ A plenty of studies focusing on the alteration in working memory and metabolic burden of mood disorder patients indicate that mood disorder patients who are complicated by obesity show greater cognitive impairment than patients with normal weight.^[29,30]^ Nevertheless, few researches have investigated the correlation between disorganized metabolites and cognitive deficits in MDD patients.

In the present study, therefore, we aimed to use metabolomics technology to identify differential metabolites in MDD and HCs, and to investigate whether the differential metabolites identified in our samples are associated with deficits in spatial working memory of MDD patients and HCs.

## 2 Methods

Permission was granted by West China Hospital of Sichuan University. All procedures in this research were designed and carried out in accordance with the guidelines issued by the Ethical Committee of Sichuan University. And all assessments were carried out after the participants and their legal gardens signed the informed consent forms.

### 2.1 Participants

We recruited 136 right-handed participants aged 18 to 60 years from the Mental Health Center of West China Hospital, Sichuan University. The participants consisted of 53 drug-naïve MDD patients and 83 sex-, gender-, and BMI-matched HCs. All patients were diagnosed as having MDD according to the Diagnostic and Statistical Manual of Mental Disorders, Fourth Edition (DSM-IV). Meanwhile, HCs were recruited via advertisements mainly in Sichuan province. The healthy volunteers were interviewed by psychiatrists to assure that none of them had a current or past history of psychiatric disorders. Participants were excluded if they had

1. endocrine diseases, metabolic disorders, or receiving hormone medication;
2. any serious physical diseases;
3. other psychiatric disorders, such as dementia, schizophrenia, and substance abuse;
4. obvious psychosocial factors; and
5. any psychotropic medications during the past 12 weeks.

### 2.2 Plasma sample detection

Peripheral blood of all participants was collected using EDTA-anticoagulated tubes on the first day of taking part in this research. The blood samples were then centrifuged at 2000 g for 5 minutes, and the upper layer was transferred to a fresh tube and stored at −80°C.

Then, the plasma samples were removed from the refrigerator and thawed in the ice bath. 0.1 ml defrosted sample was taken and added to a new 1.5 ml centrifuge tube. Mass spectrometric methanol (i.e., the volume ratio of methanol/water is 4/1) that 4 timed the volume of 0.4 ml was added to the new 1.5 ml centrifuge tube, mixed, and precipitated. After the removal of the protein, it was placed in the ice bath for 5 minutes and centrifuged at 15,000 g/minute at 4°C for 10 minutes. Finally, the above supernatant solution was diluted with mass spectroscopic water to the methanol content of 53% and placed in a centrifuge tube at 15,000 g for centrifugation at 4°C for 10 minutes. The supernatant was collected, and the sample was injected into the liquid chromatography-tandem mass spectrometry (LC-MS/MS) for analysis.

LC-MS/MS system analysis was injected with the filtrate. Then both a Vanquish UHPLC system (Thermo Fisher) and an Orbitrap Q Exactive HF-X mass spectrometer (Thermo Fisher) were used. Using the 16-minute linear gradient at a flow rate of 0.2 ml/minute, the plasma of participants was injected onto a Hyperil Gold column (100 × 2.1 mm, 1.9 μm). Both eluent A (0.1% FA in Water) and methanol B were the positive polarity mode of the eluents. The solvent gradient was set as follows: 2% B for 12.0 minutes; 100% B for 14.0 minutes; 100% to 2% B for 1.5 minutes; 2% to 100% B for 14.1 minutes; and 2% B for 16 minutes. Then we operated Q Exactive HF-X mass spectrometer in bipolarity modes with the help of capillary temperature at the temperature of 320°C, spray voltage of 3.2 kV, aux gas flow rate of 10 arb, and sheath gas flow rate of 35 arb.

### 2.3 Differential metabolites identification

Metabolites, which were obtained from LC-MS/MS system analysis, were normalized for subsequent analyses using log transformation. Two different analytical methods were used to find the distinctive metabolites for MDD.

First of all, the above metabolites were used to build the spare partial least squares-discriminant analysis model (sPLS-DA). Spare PLS-DA is a multivariate monitoring method for the classification of high dimensional bio-omics data, and it can select variables and reduce dimension.^[17]^ After log-transformed, the analysis was run with sPLS-DA, which was performed using mixOmics package with R. Then metabolites in the first component was selected and sorted by the absolute value of the variable importance in projection (VIP).^[16]^

Meanwhile, we used RF analysis to study our metabolites. RF is a non-parametric machine learning algorithm, and its internal verification steps use out-of-bag subsampling to give a fair estimate of the robustness of biomarkers. RF algorithm can avoid overfitting to some extent. In the current study, log-transformed data was analyzed using random forest package with R. Then, we sorted metabolites by the mean decrease accuracy important scores, which could help us to identify biomarkers.^[19,31]^

Finally, 5x cross validation was performed to suggest the appropriate number of metabolites that should be selected from the 2 model according to the predicted distance.

### 2.4 Neurocognitive assessments

Thirty five MDD patients and 48 HCs had completed our neurocognitive assessments. Neurocognitive function was evaluated at the first assessment of both patients and HCs using spatial working memory (SWM) task in Cambridge Neuropsychological Tests Automated Battery (CANTAB: http://www.cantab.com). The SWM task is a test that evaluates the ability to retain spatial information. In this task, the participant searched for hidden “okens” in a spatial array of colored boxes. By touching the boxes and using the elimination process, the participant should find a blue “token” in each box and fill a blank column on the right side of the screen with them. The number of boxes gradually increased to 8. In each trial, the color and position of the boxes were changed to avoid the same search strategy.^[32]^ Outcome measures consisted of 2 parts: errors and strategy. The higher the errors score, the poorer accuracy of working memory. The higher the strategy score, the less efficient task performance.^[33,24]^

Finally, the relationship between differential metabolites and neurocognition was analyzed respectively by using spearman rank correlation.

## 3 Results

### 3.1 Demographic characteristics of samples

From 2015 to 2018, we recruited 136 participants in total. Table 1 summarized the demographic data of all participants, and demographic data of participants who had completed neurocognitive test was also summarized in Table S1 (see Table S1, Supplemental Digital Content, which demonstrates the demographic data of participants who had completed neurocognitive test.). There was no significant difference in age, gender ratio, or body mass index (BMI) (*P* > .05).

**Table 1:** Demographic characteristics of participants.

Variables	MDD (n = 53)	HCs (n = 83)	*P*∗ value
Sex (male/female)	14/39	31/52	.198
Age (year)	25.25 ± 7.50	26.40 ± 8.62	.491
BMI	20.61 ± 2.71	21.29 ± 2.79	.171
HAMD total scores	21.57 ± 5.51	–	–
HAMA total scores	15.57 ± 5.86	–	–

BMI = body mass index, HAMA = Hamilton anxiety Rating Scale, HAMD = Hamilton Depression Rating Scale, HCs = healthy controls, MDD = major depressive disorder.

### 3.2 Classification of MDD by metabolite profile

Of the 728 metabolites obtained from LC-MS/MS system analysis, 296 were selected by applying a cut-off for QC samples’ coefficient of variation ≤20% (Table S2). (See Table S2, Supplemental Digital Content, which demonstrates the involved metabolites.)

**Figure 1:**
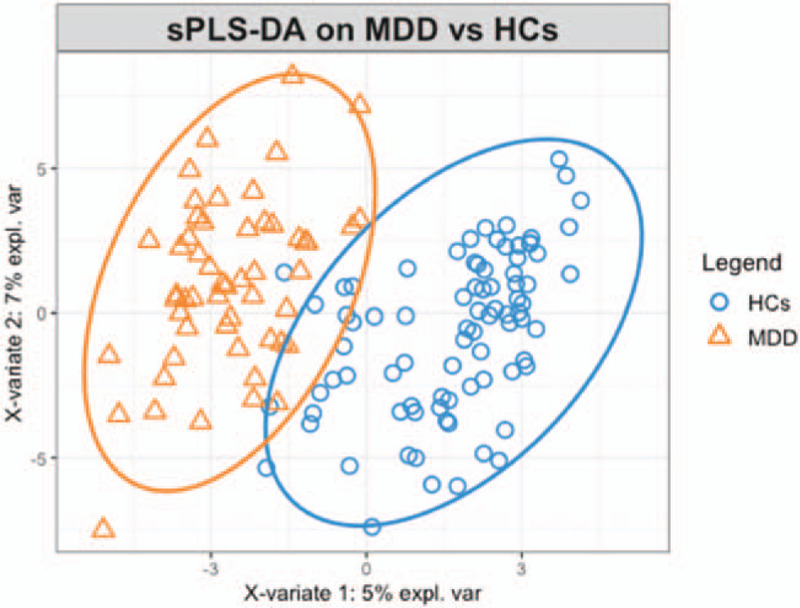
Classification of MDD and HCs using metabolites with sPLSDA analysis. MDD (yellow dots) and HCs (blue dots) were distinguished by using metabolites which were included in component 1(X-variate 1) and component 2(X-variate 2) of sPLS-DA model. The ellipse is the 95% confidence interval. HCs = healthy controls, MDD = major depressive disorder.

**Figure 2:**
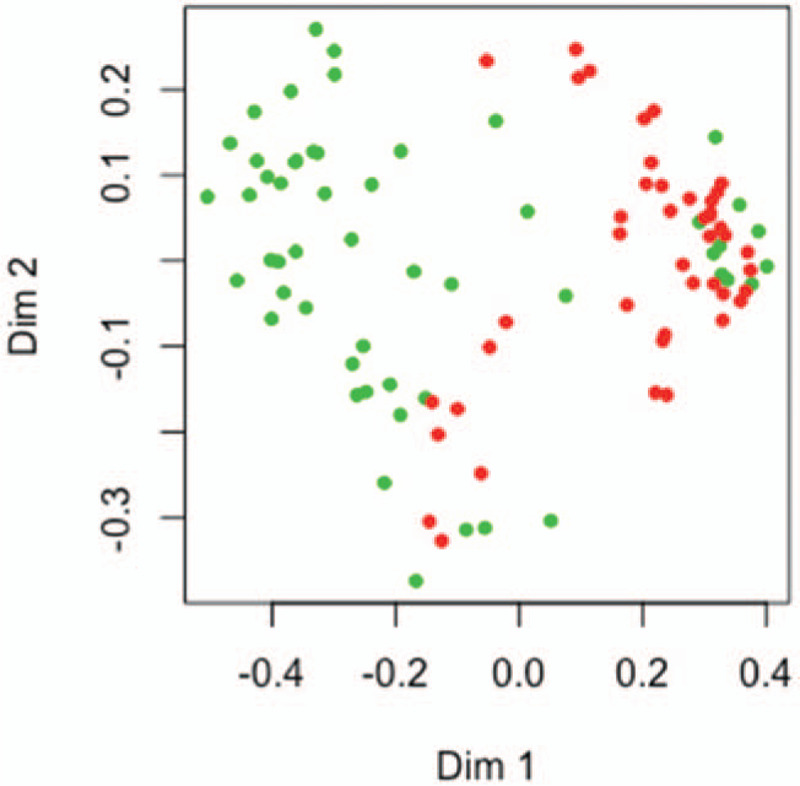
Classification of MDD and HCs using metabolites with RF analysis. Priori label information of metabolites obtained through random forest model were plotted on the first tow dimensions of the Multi-Dimensional Scaling plot. MDD samples (red dots) and HCs (green dots) were classified by that metabolic information. HCs = healthy controls, MDD = major depressive disorder.

Spare PLS-DA analysis indicated that metabolites could discriminate patients from HCs (Fig. 1). Component 1 consisted of 30 variables, and the area under the curve (AUC) of it was 0.98. Then, RF analysis was used to explore the underlying the characteristics of our data. Six hundred trees were grown, and the so-called out-of-bag estimate of error rate was 8.25%. The error-rate did not decrease with the number of trees constructed, and the AUC was 0.89. RF model could also discriminate MDD patients from controls (Fig. 2). Five-fold cross-validation was used to evaluate the stability of RF model and determine the number of variables. However, this method indicated that the more variables were included the higher coefficient of variation error was. The top 5 metabolites could be the optimal variables required by this model.

The top 5 metabolites which were found in sPLS-DA model and RF model were the same: gamma-glutamyl leucine, leucine-enkephalin, valeric acid, vanillin, and mestranol. However, only 3 metabolites showed significant differences between MDD and HCs. They are gamma-glutamyl leucine (*P* = 5.25E-08), leucine-enkephalin (*P* = 9.38E-10), and valeric acid (*P* = .002) (Table 2).

**Table 2:** Differential metabolites found in sPLS-DA and random forest.

Metabolites	VIP- absolute value	Mean Decrease Accuracy	Fold Change	*P* value^∗^
gamma-glutamyl leucine	2.91	0.02	0.44	5.25E-08^∗^
Mestranol	-2.09	0.01	1.52	.35
Leucine-enkephalin	1.98	0.02	0.72	9.38E-10^∗^
Valeric acid	1.89	0.02	0.77	.002^∗^
Vanillin	1.27	0.01	0.60	.056

Metabolites = the differential metabolites we identified in our research, VIP-absolute value = the product of the variable importance and the selection frequency in the component one of sPLS-DA, Mean Decrease Accuracy = mean accuracy decreased importance score in RF model; P-values were derived from non-parametric Mann–Whitney *U* test.

### 3.3 Neurocognitive function results

Thirty five patients and 48 HCs had completed all the neurocognitive tests. Compared to HCs group, MDD patients showed worse performance on SWM-between errors (*P* = .037) and SWM-total errors (*P* = .042). However, no significant difference was found between the 2 groups in other tests of SWM task (Table 3).

**Table 3:** Differences in cognition scores between MDD patients and HCs.

	Measurement	MDD	HCs	*P* value^∗^
SWM_BE	SWM Between errors	24.00 ± 14.97	19.72 ± 21.61	.037^∗^
SWM_WE	SWM Within errors	1.76 ± 2.57	3.42 ± 10.26	.59
SWM_DE	SWM Double errors	0.88 ± 1.47	1.83 ± 5.37	.61
SWM_TE	SWM Total errors	24.88 ± 15.49	21.38 ± 23.82	.042^∗^
SWM_Stra	SWM Strategy	33.41 ± 3.60	30.79 ± 6.01	.053

HCs = healthy controls, MDD = major depressive disorder, SWM = Spatial Working Memory. *P* values were derived from non-parametric Mann–Whitney *U* test.

### 3.4 Metabolites associated with neurocognitive function

A significant, positive correlation was determined between valeric acid and SWM-between errors (*r*: 0.482, *P* = .003), SWM-within errors (*r*: 0.395, *P* = .019), and SWM-total errors (*r*: 0.508, *P* = .002) in MDD group. However, no significant correlation was observed between other metabolites and neurocognitive function. In addition, no association was found between the 3 metabolites and spatial working memory in HCs group.

## 4 Discussion

In our study, sPLS-DA analysis and RF algorithm were performed to explore the metabolic changes in MDD patients, and they both showed remarkable discrimination between MDD patients and HCs individuals with AUCs of 0.98 and 0.89. Then 5 metabolites were found to decrease in depressive individuals compared to HCs, and 3 of these metabolites showed significant differences between 2 groups, namely, gamma-glutamyl leucine, leucine-enkephalin, valeric acid. Finally, MDD patients performed worse in spatial working memory, and valeric acid were correlated with the dysfunction in spatial working memory of MDD group.

Abnormality of gamma-glutamyl leucine was identified in MDD patients for the first time. Gamma-glutamyl leucine is a dipeptide composed of gamma-glutamate and leucine.^[34]^ Low level of gamma-glutamyl leucine which is one of gamma-glutamyl amino acids reflects low glutathione (GSH) turnover,^[35,36]^ and GSH as a main protective antioxidant plays an important role in oxidant antioxidant balance.^[37]^ A study on abnormal GSH in postmortem prefrontal cortex of psychiatric patients showed that both GSH and an enzyme utilizing GSH were decreased in brain of MDD patients, but the rate-limiting enzyme glutamyl-cysteine ligase had no difference between MDD patients and HCs.^[38]^ These evidences suggested that the GSH depletion in MDD may not have resulted from dysfunction in oxidative stress but made patients more susceptible to oxidative damages. In addition, GSH metabolism is also a special branch of glycine, serine, and threonine metabolism.^[39]^ Previous researches have shown that abnormality in glycine, serine, and threonine metabolism could induce both affective and cognitive disorders by decreasing neuroplasticity.^[40,41]^

Leucine-enkephalin is one of enkephalin pentapeptides which are a group of endogenous neuropeptides and have antinociceptive ability in both central and peripheral nervous systems.^[42,43]^ The dysfunction of enkephalin-containing neurons was found throughout the brain of depression-like mice, and the low level of leucine-enkephalin was repeatedly reported in hippocampus, hypothalamus, and striatum of depression-like rodent models.^[44–46]^ The naturally occurring leucine-enkephalin has high affinity for delta-opioid receptors,^[47]^ and depression-like behaviors are found in delta-opioid receptors-null mice.^[48]^ A delta-opioid receptor agonist can increase the expression of the brain-derived neurotrophic factor (BDNF) mRNA and modulate neuronal plasticity.^[49,50]^ However, evidences from human samples are still lacking. In recent years, modulation of endogenous opioid tone as one of these emerging treatment-targets of MDD has showed great potential in the treatment of MDD.^[51,43]^ Our present research may provide theoretical support for these studies.

Valeric acid is mainly found in gut bacterial communities, and it is the main metabolic end product of oscillibacter, which is also found decreased in MDD patients.^[52]^ Previous studied showed that the alteration of gut microbiota composition in MDD patients was associated with dysfunction in neurotransmitter metabolism and low-grade inflammation via the brain-gut-microbiota axis.^[53]^ Valeric acid is capable of protecting the gastrointestinal tract function and intestinal epithelial integrity.^[54]^ Studies showed that chronic gastrointestinal inflammation could induce depression and anxiety-like behaviors in mice and that decreased BDNF mRNA was also detected in depression-like mice models.^[55]^ Valeric acid is a kind of short-chain fatty acids known for their anti-inflammatory properties. Further exploration in rodent models suggested that short-chain fatty acids could decrease the pro-inflammatory cytokine synthesis and increase the anti-inflammatory cytokine secretion at the same time.^[56,57]^ And chronic low-grade inflammation in body and alteration in cytokines which are related to inflammation had proven to be one of the etiological factors of MDD.^[58]^ In addition, valeric acid appears to be a similar structure to neurotransmitter GABA. Low level of valeric acid can increase the degradation of GABA and decline the response of GABA-A receptor, which has been proved to be a possible acting site for rapid treatment in MDD.^[59,60]^ In addition, valeric acid is also known as an N-methyl-D-aspartate receptors (NMDARs) antagonist,^[61]^ and the abnormality in NMDARs activity can result in both affective and cognitive disorders.^[41]^

In our study, MDD group showed worse spatial working memory than HCs patients and had significantly higher scores on SWM-between errors and SWM-total errors. We found MDD patients performed worse in SWM-errors but not strategy-using, which suggested that neurocognitive function was mainly damaged in “pure” working memory. Our study results were consistent with those focusing on the association between neurocognition and MDD.^[24]^ Previous researches had demonstrated that poor performance in SWM task was sensitive to frontal lobe damage. A network-based analysis of resting state functional connectivity in MDD showed that the abnormality of executive control network in brain was correlated with emotional processing and behavioral disorder,^[62]^ and the dorsolateral prefrontal cortex in the executive control network showed decreased connectivity with other regions.^[63]^ Furthermore, hemodynamic abnormality and grater microvascular dysfunction in frontal lobe had also been found in acute episodes of depression.^[64,65]^

In our study, we found a significantly positive correlation between valeric acid and working memory in MDD patients, including SWM-between errors, SWM-within errors, and SWM-total errors. Data from dementia rodent models have demonstrated that valeric acid could inactivate alpha-ketoglutarate dehydrogenase and then elevate the level of GABA which could ameliorate memory.^[66]^ In addition, preclinical evidences have showed that the inhibition of the glycine site of NMDARs could alleviate damage in cognitive function.^[41]^ Recently, increasing literatures reveled that valeric acid had a promising blood pressure lowering function.^[67]^ Valeric acid can penetrate rapidly from the colon to blood and then brain, and it is also found to inhibit the intrarenal renin-angiotensin system and interfere with the sympathetic nervous system via G-protein coupled receptors.^[68,69]^ Hence, we suggested that decreasing level of valeric acid in MDD individuals could contribute to hemodynamic dysfunction in multiple regions in brain, affecting neurocognitive function including working memory. However, in MDD patients, we found high level of valeric acid indicated better performance in SWM-errors test, we therefore suspected that valeric acid may be a protect factor for neurocognition when inflammation had attacked MDD patients’ immune and metabolic system, and relatively high level of valeric acid in patients may help to modulate hemodynamics. However, more neurocognitive assessment should be applied to validate our results.

Despite the findings, our study also has various limitations that need to be considered. First, we did not control the fasting time of the enrolled participants precisely, but we did complete the blood collection of all participants at 4 pm, and we assume that most participants had fasted at least longer than 2 hours at that time. Second, both patients and HCs’ BMI were recorded, and there was not significant difference between 2 groups, but dietary habits of participants may also affect our results. Further studies better take the influence of dietary habits into consideration. Then, only plasma samples were collected in our study, other samples like cerebrospinal fluid and urine could also be the selection to validate our results. Finally, the size of participants which we recruited were relatively small, larger sample sizes are needed to verify our results. Therefore, future researches should take those factors into account when identifying differential metabolites in MDD.

## 5 Conclusions

In conclusion, 2 algorithm-sPLS-DA and RF analysis methods helped us to identify differential metabolites. Three metabolites were proven to be involved in the pathophysiological process of MDD, namely, gamma-glutamyl leucine, leucine-enkephalin, valeric acid. MDD patients showed worse working memory than HCs, and dysfunction in working memory of MDD individuals had a significant association with valeric acid.

## Author contributions

**Formal analysis:** Yue Du, Jinxue Wei.

**Funding acquisition:** Xiaohong Ma.

**Investigation:** Xiao Yang, Yikai Dou, Liansheng Zhao, Xueyu Qi, Xueli Yu.

**Project administration:** Wanjun Guo, Qiang Wang, Wei Deng, Minli Li, Tao Li, Xiaohong Ma.

**Supervision:** Tao Li, Xiaohong Ma.

**Writing – original draft:** Yue Du.

**Writing – review & editing:** Yue Du, Jinxue Wei, Dongtao Lin.

## Supplementary Material

Supplemental Digital Content

## Supplementary Material

Supplemental Digital Content
