# Supplementary material for: Plasma metabolites were associated with spatial working memory in major depressive disorder
Source: Medicine (Baltimore). 2021 Feb 26;100(8):e24581. doi: 10.1097/MD.0000000000024581 (PMC7909221; doi:10.1097/MD.0000000000024581)
Supplement: Supplemental Digital Content [file medi-100-e24581-s001.pdf]

Table S1. Demographic characteristics of participants who had completed neurocognitive assessment.

Variables	MDD (n=35)	HCs (n=48)	P-value*
Sex (male/female)	9/26	19/29	0.242
Age (year)	24.34±6.53	26.90±8.89	0.188
BMI	20.25±2.44	21.23±2.59	0.114
HAMD total scores	14.51±5.25	-	-
HAMA total scores	21.23±5.23	-	-
IQ test	110.51±14.35	113.23±12.63	0.382

HCs: healthy controls; MDD: major depressive disorder; HAMD: Hamilton Depression Rating Scale; HAMA: Hamilton anxiety Rating Scale; BMI: body mass index; IQ test: the total scores of Wechsler Adult Intelligence Scale – Revised in China (WAIS-RC).
