# Supplementary material for: Plasma metabolites were associated with spatial working memory in major depressive disorder
Source: Medicine (Baltimore). 2021 Feb 26;100(8):e24581. doi: 10.1097/MD.0000000000024581 (PMC7909221; doi:10.1097/MD.0000000000024581)
Supplement: Supplemental Digital Content [file medi-100-e24581-s002.docx]

| Table S2. Complete list of metabolites in major depressive disorder patients and healthy controls | | | | |
| --- | --- | --- | --- | --- |
| metabolites | sPLS-DA.rank | sPLS-DA.-vip | RF.rank | RF.vip |
| Leucine-enkephalin | 3 | 1.9788489 | 1 | 0.021559662 |
| Gamma-Glu-Leu | 1 | 2.93005242 | 2 | 0.021320268 |
| Valeric acid | 4 | 1.91544937 | 3 | 0.017172976 |
| Mestranol | 2 | -2.0778431 | 4 | 0.011471513 |
| Vanillin | 5 | 1.29291188 | 5 | 0.011243262 |
| 3b,7b-Dihydroxy-5-androsten-17-one | 11 | -0.434418 | 6 | 0.007973243 |
| Nervonic acid | 8 | -0.8698528 | 7 | 0.007768239 |
| O-Desmethylvenlafaxine | 13 | -0.2714645 | 8 | 0.007050403 |
| Tetrahydrocorticosterone | 12 | 0.43166062 | 9 | 0.006997853 |
| 3-Acetyl-2,5-dimethylfuran | 6 | 1.29291188 | 10 | 0.00673405 |
| 1-Methylguanidine |  | 0 | 11 | 0.005959484 |
| 1,3-Dimethyluracil | 7 | 1.02326372 | 12 | 0.005486729 |
| N-Desmethyltramadol | 14 | -0.2235286 | 13 | 0.004999554 |
| Neopterin | 9 | -0.8682529 | 14 | 0.004843771 |
| Lauric acid | 24 | -0.0242596 | 15 | 0.004547876 |
| L-Aspartic acid | 10 | 0.81094807 | 16 | 0.00450641 |
| 13-Hpotre(R) | 21 | -0.0498473 | 17 | 0.004502692 |
| N-benzyl-N-isopropyl-N'-[4-(trifluoromethoxy)phenyl]urea | 15 | -0.2054875 | 18 | 0.003943061 |
| Albendazole sulfoxide | 23 | 0.03338709 | 19 | 0.003335973 |
| 2-Deoxy-D-galactose |  | 0 | 20 | 0.003087119 |
| 1,2,3-Trihydroxybenzene | 16 | -0.1400049 | 21 | 0.003067051 |
| Hydroxypyruvic acid |  | 0 | 22 | 0.002992016 |
| Rosmarinic Acid | 27 | 0.00719158 | 23 | 0.002554296 |
| Acetylenedicarboxylic acid |  | 0 | 24 | 0.002473199 |
| FAHFA (16:0/14:1) | 25 | -0.0092568 | 25 | 0.002088256 |
| 4'-Chloro-α-pyrrolidinopropiophenone |  | 0 | 26 | 0.002006803 |
| 5-hydroxy-4-methoxy-5,6-dihydro-2H-pyran-2-one | 22 | -0.0494742 | 27 | 0.001931861 |
| Asparagine | 17 | 0.13912331 | 28 | 0.001847521 |
| 3-Methyladipic acid |  | 0 | 29 | 0.00165724 |
| Pyruvic acid | 18 | 0.12014099 | 30 | 0.001560197 |
| 1-Methyluric acid |  | 0 | 31 | 0.001552692 |
| 1-Naphthol |  | 0 | 32 | 0.001402219 |
| 2-Hydroxycinnamic acid | 20 | 0.0640368 | 33 | 0.0013702 |
| Gibberellic acid |  | 0 | 34 | 0.001321614 |
| Asp-Phe methyl ester |  | 0 | 35 | 0.001284006 |
| Ferulic acid |  | 0 | 36 | 0.00126024 |
| N-Acetyl-aspartic acid | 19 | 0.07603839 | 37 | 0.001245022 |
| LPA 20:4 |  | 0 | 38 | 0.001180242 |
| Testosterone sulfate |  | 0 | 39 | 0.00117345 |
| LPE 20:4 |  | 0 | 40 | 0.000960436 |
| Glycyrrhizic acid |  | 0 | 41 | 0.000937448 |
| D-Glucoronic Acid |  | 0 | 42 | 0.000920052 |
| D-(-)-Quinic acid |  | 0 | 43 | 0.00091595 |
| 2,3,4,9-Tetrahydro-1H-β-carboline-3-carboxylic acid | 29 | 0.00085796 | 44 | 0.000903263 |
| Melatonin |  | 0 | 45 | 0.000884093 |
| Isoliquiritigenin |  | 0 | 46 | 0.000816915 |
| Gluconic acid |  | 0 | 47 | 0.000792837 |
| LPG 18:2 |  | 0 | 48 | 0.000788159 |
| Carbaprostacyclin |  | 0 | 49 | 0.000781196 |
| 4-Methylumbelliferone hydrate |  | 0 | 50 | 0.000746046 |
| δ-Ribono-1,4-lactone |  | 0 | 51 | 0.000727787 |
| D-(+)-Malic acid | 28 | 0.00195647 | 52 | 0.000680266 |
| Androsterone glucuronide |  | 0 | 53 | 0.00066119 |
| 2-Arachidonoyl glycerol |  | 0 | 54 | 0.000646606 |
| Creatinine |  | 0 | 55 | 0.000644444 |
| 18-β-Glycyrrhetinic acid |  | 0 | 56 | 0.000632601 |
| 4-Hydroxy-6-methyl-2-pyrone |  | 0 | 57 | -0.000629699 |
| 4-Phenylbutyric acid |  | 0 | 58 | 0.000604056 |
| Jasmonic acid |  | 0 | 59 | 0.000575155 |
| 4-Pyridoxic acid |  | 0 | 60 | 0.000562544 |
| Tetradecanedioic acid |  | 0 | 61 | 0.000528071 |
| Dl-3,4-Dihydroxymandelic Acid |  | 0 | 62 | 0.000500374 |
| 4-Hydroxybutyric acid (GHB) | 26 | 0.00738426 | 63 | 0.000491407 |
| Taurine |  | 0 | 64 | 0.00047246 |
| Dimethyl fumarate |  | 0 | 65 | 0.000471632 |
| (2S)-4-Oxo-2-phenyl-3,4-dihydro-2H-chromen-7-yl beta-D-glucopyranoside |  | 0 | 66 | 0.000462106 |
| PC (5:0/13:1) |  | 0 | 67 | -0.000451422 |
| Mesaconic acid |  | 0 | 68 | 0.000428664 |
| Acrylic acid |  | 0 | 69 | -0.00042103 |
| 3-Hydroxy-3-methylbutanoic acid |  | 0 | 70 | -0.000385851 |
| Threonine |  | 0 | 71 | -0.000378753 |
| (+/-)-Equol |  | 0 | 72 | 0.000374742 |
| Artemisinin |  | 0 | 73 | -0.000362771 |
| Lysine |  | 0 | 74 | -0.000358029 |
| N-Methylisoleucine |  | 0 | 75 | 0.000357291 |
| 1,2-Dihydro-1,2-naphthalenediol |  | 0 | 76 | 0.000354957 |
| D-Alanyl-D-Alanine |  | 0 | 77 | 0.000349032 |
| Dl-Proline |  | 0 | 78 | 0.000345146 |
| Epinephrine |  | 0 | 79 | 0.00034263 |
| Piperine |  | 0 | 80 | 0.000333309 |
| Citric acid |  | 0 | 81 | -0.000333307 |
| Gly-Phe |  | 0 | 82 | 0.000333299 |
| Glycoursodeoxycholic acid |  | 0 | 83 | 0.00032931 |
| O-Feruloyl Quinic Acid |  | 0 | 84 | 0.000327214 |
| Cholic acid |  | 0 | 85 | 0.000321732 |
| DL-α-Aminocaprylic acid |  | 0 | 86 | 0.000321089 |
| Docosahexaenoic acid |  | 0 | 87 | -0.000309798 |
| 1,5-Anhydro-D-glucitol |  | 0 | 88 | 0.000302741 |
| PE (14:0e/2:0) |  | 0 | 89 | 0.00030195 |
| Cortisone |  | 0 | 90 | 0.000298891 |
| PC (14:1e/4:0) |  | 0 | 91 | 0.000298456 |
| Azelaic acid |  | 0 | 92 | 0.000296744 |
| 9-Hydroxy-(10E,12Z,15Z)-Octadecatrienoic Acid |  | 0 | 93 | 0.000294781 |
| PC (15:1/15:1) |  | 0 | 94 | 0.000293213 |
| Ginkgoic acid |  | 0 | 95 | 0.000292664 |
| Hydrocortisone |  | 0 | 96 | 0.000289841 |
| Gamma-Caprolactone |  | 0 | 97 | -0.000289199 |
| 4-Methoxybenzaldehyde |  | 0 | 98 | -0.000289152 |
| Oleoyl-L-α-lysophosphatidic acid |  | 0 | 99 | 0.000288211 |
| 4-Methoxycinnamic acid |  | 0 | 100 | 0.000285215 |
| LPG 16:1 |  | 0 | 101 | 0.000284168 |
| Lysops 22:6 |  | 0 | 102 | 0.000280295 |
| Hydroquinone |  | 0 | 103 | -0.000278286 |
| Flavanone |  | 0 | 104 | -0.000276806 |
| Prostaglandin G2 |  | 0 | 105 | 0.0002722 |
| Dl-Tropic acid |  | 0 | 106 | 0.00027135 |
| Picolinic acid |  | 0 | 107 | 0.00026998 |
| 2-(3,4-dimethoxyphenyl)ethanamine |  | 0 | 108 | 0.000267319 |
| Lysopc 16:0 (2N Isomer) |  | 0 | 109 | 0.000265332 |
| O-Desmethylnaproxen |  | 0 | 110 | 0.000260628 |
| Glycocholic acid |  | 0 | 111 | 0.000253444 |
| (5ξ,9ξ)-17-Hydroxykaur-15-en-19-oic acid |  | 0 | 112 | 0.000243745 |
| Acetyl-L-carnitine |  | 0 | 113 | 0.000242812 |
| 9-Oxo-10(E),12(E)-octadecadienoic acid |  | 0 | 114 | -0.000241289 |
| Indole-3-butyric acid |  | 0 | 115 | 0.000239584 |
| SM (d14:2/20:0) |  | 0 | 116 | 0.000238818 |
| DL-Stachydrine |  | 0 | 117 | -0.000236856 |
| Ascorbic acid |  | 0 | 118 | 0.00023341 |
| Cytidine 5'-Monophosphate-N-Acetylneuraminic Acid |  | 0 | 119 | 0.000230613 |
| Pyrogallol |  | 0 | 120 | 0.000228798 |
| 9-Hpode |  | 0 | 121 | -0.000228087 |
| Dl-Lanthionine |  | 0 | 122 | 0.000227905 |
| Coniferaldehyde |  | 0 | 123 | 0.000225109 |
| 1-(3,4-dimethoxyphenyl)ethan-1-one oxime |  | 0 | 124 | -0.000222606 |
| Ribitol(Adonitol) |  | 0 | 125 | -0.000221795 |
| Mesterolone |  | 0 | 126 | 0.000219346 |
| α-Eleostearic acid |  | 0 | 127 | -0.000211303 |
| L-Homoarginine |  | 0 | 128 | -0.000205969 |
| Vanillyl alcohol |  | 0 | 129 | 0.00020584 |
| Docosatrienoic acid |  | 0 | 130 | 0.000204389 |
| N-Phenylacetylglutamine |  | 0 | 131 | 0.000204082 |
| Estrone |  | 0 | 132 | -0.000202177 |
| Noroxycodone-d3 |  | 0 | 133 | -0.000199712 |
| Taurocholic Acid |  | 0 | 134 | -0.000198879 |
| L-Histidine |  | 0 | 135 | -0.000196849 |
| Proline |  | 0 | 136 | -0.000195536 |
| Riboflavin B2 |  | 0 | 137 | 0.000194741 |
| 7-Ketodeoxycholic acid |  | 0 | 138 | 0.000192187 |
| Methoxyacetyl fentanyl-d5 |  | 0 | 139 | 0.000192183 |
| cis,cis-Muconic acid | 30 | -5.571E-05 | 140 | 0.000184836 |
| dGDP(2'-Deoxyguanosine-5'-diphosphate) |  | 0 | 141 | -0.000184625 |
| Benzamide |  | 0 | 142 | -0.000183495 |
| D-(-)-Glutamine |  | 0 | 143 | 0.000183418 |
| N-cyclooctylurea |  | 0 | 144 | 0.00018277 |
| 3,4-Dihydroxybenzaldehyde |  | 0 | 145 | -0.000176821 |
| aminoimidazole carboxamide ribonucleotide |  | 0 | 146 | -0.000176668 |
| 4-oxo-4-(5,6,7,8-tetrahydronaphthalen-1-ylamino)but-2-enoic acid |  |  | 147 | 0.000170979 |
| Taurochenodeoxycholic acid |  | 0 | 148 | -0.000162564 |
| Taurolithocholic acid 3-sulfate |  | 0 | 149 | -0.000161078 |
| Adipamide |  | 0 | 150 | 0.000159248 |
| L-Tyrosinemethylester |  | 0 | 151 | -0.000158463 |
| Decanoylcarnitine |  | 0 | 152 | -0.000158278 |
| Indole-3-carbinol |  | 0 | 153 | -0.000157402 |
| Sucrose |  | 0 | 154 | 0.00015711 |
| Gramine |  | 0 | 155 | -0.000151414 |
| IMP |  | 0 | 156 | -0.000151104 |
| 1,6-Bis-O-[(2E)-3-(4-hydroxyphenyl)-2-propenoyl]-β-D-glucopyranose |  | 0 | 157 | 0.000148316 |
| Capryloylglycine |  | 0 | 158 | 0.000145998 |
| 6-Methylquinoline |  | 0 | 159 | 0.000142537 |
| LPI 18:2 |  | 0 | 160 | 0.000138974 |
| 2'-Hydroxy-5'-methylacetophenone |  | 0 | 161 | 0.000138968 |
| Lysopc 18:2 |  | 0 | 162 | 0.000138228 |
| D-Methionine |  | 0 | 163 | -0.000137719 |
| D-Panthenol |  | 0 | 164 | 0.000136482 |
| 6-Keto-prostaglandin f1alpha |  | 0 | 165 | -0.000134061 |
| LPI 20:3 |  | 0 | 166 | 0.000131619 |
| Tyramine |  | 0 | 167 | -0.000131296 |
| L-Alanyl-L-leucine |  | 0 | 168 | -0.000130187 |
| 8Z,11Z,14Z-Eicosatrienoic acid |  | 0 | 169 | -0.000130069 |
| malonyl-CoA |  | 0 | 170 | -0.000127923 |
| Indole-3-acrylic acid |  | 0 | 171 | -0.000127197 |
| PC (18:4e/18:5) |  | 0 | 172 | 0.000124622 |
| LPG 16:0 |  | 0 | 173 | 0.000123365 |
| 5-Methoxyindole-3-Carbaldehyde |  | 0 | 174 | 0.000123299 |
| Methyl indole-3-acetate |  | 0 | 175 | 0.000122829 |
| 13,14-dihydro-15-keto-PGD2 |  | 0 | 176 | 0.00012213 |
| DL-3,4-Dihydroxyphenyl glycol |  | 0 | 177 | -0.00012166 |
| LPI 16:0 |  | 0 | 178 | -0.000120911 |
| LPI 20:4 |  | 0 | 179 | 0.000120846 |
| N-Acetylthreonine |  | 0 | 180 | -0.000120816 |
| cis-4-Hydroxy-D-proline |  | 0 | 181 | -0.000118509 |
| Cortisol |  | 0 | 182 | 0.000118127 |
| cholesteryl sulfate |  | 0 | 183 | 0.000116555 |
| D-Glucarate |  | 0 | 184 | -0.00011644 |
| 4-Phenylbutazone |  | 0 | 185 | -0.000115957 |
| (5-L-Glutamyl)-L-Amino Acid |  | 0 | 186 | -0.000114687 |
| LPI 18:1 |  | 0 | 187 | -0.000114499 |
| L-Ascorbate |  | 0 | 188 | -0.000114343 |
| Dihydrothymine |  | 0 | 189 | 0.000114217 |
| Elaidic acid |  | 0 | 190 | -0.000113388 |
| ACar 13:0 |  | 0 | 191 | -0.000113078 |
| Cinnamoylglycine |  | 0 | 192 | -0.000112172 |
| O-Anisic Acid |  | 0 | 193 | 0.000111842 |
| Propylparaben |  | 0 | 194 | -0.000111292 |
| Lysopc 18:3 |  | 0 | 195 | 0.000110917 |
| Docosapentaenoic acid |  | 0 | 196 | 0.000106753 |
| (2E)-3-(3,4-dimethoxyphenyl)prop-2-enoic acid |  | 0 | 197 | -0.000106219 |
| 2-(4-hydroxy-1,3-thiazol-2-yl)-1-phenylethan-1-one |  | 0 | 198 | 0.000104447 |
| Lysopg 18:1 |  | 0 | 199 | -0.000103374 |
| 17-α-Methyltestosterone |  | 0 | 200 | -9.47267E-05 |
| LPC 22:6 |  | 0 | 201 | 9.41225E-05 |
| Crotonic acid |  | 0 | 202 | 9.20324E-05 |
| Beta-Alanine |  | 0 | 203 | -9.1744E-05 |
| LPE 18:1 |  | 0 | 204 | -9.0169E-05 |
| DL-α-Methoxyphenylacetic acid |  | 0 | 205 | -8.9899E-05 |
| Hydroxytriazolam |  | 0 | 206 | 8.92157E-05 |
| Octadecanamine |  | 0 | 207 | 8.9037E-05 |
| 5-Aminovaleric acid |  | 0 | 208 | 8.76244E-05 |
| Picolinamide |  | 0 | 209 | -8.63636E-05 |
| acetoacetate |  | 0 | 210 | -8.53195E-05 |
| Thymine |  | 0 | 211 | -8.48378E-05 |
| Isoquinoline |  | 0 | 212 | -8.43221E-05 |
| Isobutyric acid |  | 0 | 213 | -8.36417E-05 |
| N-acetyl-L-ornithine |  | 0 | 214 | 8.32759E-05 |
| Estrone sulfate |  | 0 | 215 | -8.27736E-05 |
| A-Ketoglutaric Acid |  | 0 | 216 | -8.26823E-05 |
| ACar 12:3 |  | 0 | 217 | -8.14815E-05 |
| Dl-Mevalonolactone |  | 0 | 218 | -8.07823E-05 |
| Decanoic acid |  | 0 | 219 | -8.01282E-05 |
| Oleamide |  | 0 | 220 | 7.96926E-05 |
| Lysopc 16:1 |  | 0 | 221 | 7.92234E-05 |
| Taurochenodesoxycholic Acid |  | 0 | 222 | -7.87829E-05 |
| Androstenedione |  | 0 | 223 | 7.86697E-05 |
| methyl {[(2-oxo-2H-pyran-6-yl)carbonyl]amino}methanethioate |  | 0 | 224 | 7.84314E-05 |
| Linoleic acid |  | 0 | 225 | -7.84314E-05 |
| D-(+)-Maltose |  | 0 | 226 | -7.77305E-05 |
| Phenylacetaldehyde |  | 0 | 227 | 7.66247E-05 |
| 4-(allyloxy)-1,2-dihydroquinolin-2-one |  | 0 | 228 | 7.65306E-05 |
| Arachidonic acid |  | 0 | 229 | 7.62527E-05 |
| LPI 16:1 |  | 0 | 230 | 7.61441E-05 |
| γ-Aminobutyric Acid |  | 0 | 231 | 7.48988E-05 |
| Phenylpyruvic Acid |  | 0 | 232 | -7.47981E-05 |
| PC (18:3e/2:0) |  | 0 | 233 | -7.43397E-05 |
| N-Phenylacetylglycine |  | 0 | 234 | 7.43123E-05 |
| All-Trans-13,14-Dihydroretinol |  | 0 | 235 | 7.35059E-05 |
| D-(-)-Mannitol |  | 0 | 236 | 7.21538E-05 |
| 3-O-β-D-Glucopyranosylandrographolide |  | 0 | 237 | 6.98347E-05 |
| D-Xylonic Acid Lithium Salt |  | 0 | 238 | -6.4002E-05 |
| 3-Pyridinol |  | 0 | 239 | 6.17597E-05 |
| D-Arabinose |  | 0 | 240 | -5.7922E-05 |
| Lauric acid ethyl ester |  | 0 | 241 | -5.73945E-05 |
| 2-Hydroxyvaleric acid |  | 0 | 242 | 5.66302E-05 |
| N-Desmethylvenlafaxine |  | 0 | 243 | 5.58637E-05 |
| 3-Methylglutaric acid |  | 0 | 244 | -5.55498E-05 |
| LPC 22:4 |  | 0 | 245 | 5.34121E-05 |
| Glycyl-L-Proline |  | 0 | 246 | -5.30945E-05 |
| Trolox |  | 0 | 247 | 5.06304E-05 |
| Guvacoline |  | 0 | 248 | 4.90486E-05 |
| L-Cysteine-glutathione gisulfide |  | 0 | 249 | 4.89191E-05 |
| Neosaxitoxin |  | 0 | 250 | 4.70357E-05 |
| L-Homocitrulline |  | 0 | 251 | -4.65587E-05 |
| Adrenic acid |  | 0 | 252 | 4.54545E-05 |
| 4-Hydroxybenzylcyanide |  | 0 | 253 | -4.54052E-05 |
| DL-Lysine |  | 0 | 254 | -4.34783E-05 |
| LPC 20:1 |  | 0 | 255 | -4.34508E-05 |
| Dl-Threitol |  | 0 | 256 | 4.197E-05 |
| Pilocarpine |  | 0 | 257 | 4.17814E-05 |
| Styrene |  | 0 | 258 | -4.15184E-05 |
| LPA 16:0 |  | 0 | 259 | -4.12778E-05 |
| Indole-5-Carboxylic Acid (98%) |  | 0 | 260 | 4.08824E-05 |
| Taurochenodeoxycholate |  | 0 | 261 | 4.02336E-05 |
| Uric acid |  | 0 | 262 | -0.00004 |
| Eicosapentaenoic acid |  | 0 | 263 | -3.92191E-05 |
| (±)-Abscisic acid |  | 0 | 264 | 3.92157E-05 |
| Tetrahydrocortisone |  | 0 | 265 | 3.8708E-05 |
| DL-Tryptophan |  | 0 | 266 | -3.61792E-05 |
| 10-Hydroxydecanoic acid |  | 0 | 267 | -3.61374E-05 |
| 7-Methylguanosine |  | 0 | 268 | 3.55391E-05 |
| 16-Hydroxyhexadecanoic acid |  | 0 | 269 | 3.52347E-05 |
| N-(5-acetamidopentyl)acetamide |  | 0 | 270 | -3.36859E-05 |
| (3β,5ξ,9ξ)-3,23-Dihydroxy-1-oxoolean-12-en-28-oic acid |  | 0 | 271 | -3.33525E-05 |
| D(+)-Phenyllactic acid |  | 0 | 272 | -3.27879E-05 |
| O-Benzyl-L-tyrosine |  | 0 | 273 | -3.26832E-05 |
| Cryptotanshinone |  | 0 | 274 | 3.23466E-05 |
| N-Caffeoyl Putrescine |  | 0 | 275 | 3.22818E-05 |
| Propionylcarnitine |  | 0 | 276 | -3.20459E-05 |
| Isophorone |  | 0 | 277 | 3.05461E-05 |
| 3,4,5-trihydroxy-6-methyloxan-2-yl 2-(methylamino)benzoate |  | 0 | 278 | 3.04334E-05 |
| 20-Carboxy-Leukotriene B4 |  | 0 | 279 | 2.94376E-05 |
| S-Adenosyl-methionine |  | 0 | 280 | 2.93119E-05 |
| P-Aminobenzoate |  | 0 | 281 | -2.6428E-05 |
| 11,12-Epoxy-(5Z,8Z,11Z)-icosatrienoic acid |  | 0 | 282 | 2.45427E-05 |
| Pregnenolone |  | 0 | 283 | -2.19849E-05 |
| 1,4-Dihydro-1-Methyl-4-Oxo-3-Pyridinecarboxamide |  | 0 | 284 | -2.12652E-05 |
| 3-(pyrazin-2-ylamino)-2-(2-thienylcarbonyl)acrylonitrile |  | 0 | 285 | -2.0155E-05 |
| Tyrosylalanine |  | 0 | 286 | -1.98497E-05 |
| Betaine |  | 0 | 287 | -1.45625E-05 |
| 2-(Formylamino)Benzoic Acid |  | 0 | 288 | -1.33266E-05 |
| N1-(3-amino-4-chlorophenyl)-2-[2,4-di(tert-pentyl)phenoxy]acetamide |  | 0 | 289 | 9.74026E-06 |
| Uridine diphosphate-N-acetylglucosamine |  | 0 | 290 | -8.77578E-06 |
| PC (16:0e/2:0) |  | 0 | 291 | -7.7616E-06 |
| Calcitriol |  | 0 | 292 | -7.09504E-06 |
| Deoxycholic acid |  | 0 | 293 | 4.20537E-06 |
| L-Serine |  | 0 | 294 | 2.98984E-06 |
| γ-Linolenic acid ethyl ester |  | 0 | 295 | -2.45098E-06 |
| Delta-Tridecalactone |  | 0 | 296 | 0 |
